# Characterization of the diverse plasmid pool harbored by the *bla*_NDM-1_-containing *Acinetobacter bereziniae* HPC229 clinical strain

**DOI:** 10.1371/journal.pone.0220584

**Published:** 2019-11-19

**Authors:** Marco Brovedan, Guillermo D. Repizo, Patricia Marchiaro, Alejandro M. Viale, Adriana Limansky

**Affiliations:** Instituto de Biología Molecular y Celular de Rosario (IBR), Departamento de Microbiología, Facultad de Ciencias Bioquímicas y Farmacéuticas, CONICET, Universidad Nacional de Rosario (UNR), Rosario, Argentina; Zhejiang University, CHINA

## Abstract

*Acinetobacter bereziniae* is an environmental microorganism with increasing clinical incidence, and may thus provide a model for a bacterial species bridging the gap between the environment and the clinical setting. *A*. *bereziniae* plasmids have been poorly studied, and their characterization could offer clues on the causes underlying the leap between these two different habitats. Here we characterized the whole plasmid content of *A*. *bereziniae* HPC229, a clinical strain previously reported to harbor a 44-kbp plasmid, pNDM229, conferring carbapenem and aminoglycoside resistance. We identified five extra plasmids in HPC229 ranging from 114 to 1.3 kbp, including pAbe229-114 (114 kbp) encoding a MOB_P111_ relaxase and carrying heavy metal resistance, a bacteriophage defense BREX system and four different toxin-antitoxin (TA) systems. Two other replicons, pAbe229-15 (15.4 kbp) and pAbe229-9 (9.1 kbp), both encoding MOB_Q1_ relaxases and also carrying TA systems, were found. The three latter plasmids contained *Acinetobacter* Rep_3 superfamily replication initiator protein genes, and functional analysis of their transfer regions revealed the mobilizable nature of them. HPC229 also harbors two smaller plasmids, pAbe229-4 (4.4 kbp) and pAbe229-1 (1.3 kbp), the former bearing a ColE1-type replicon and a TA system, and the latter lacking known replication functions. Comparative sequence analyses against deposited *Acinetobacter* genomes indicated that the above five HPC229 plasmids were unique, although some regions were also present in other of these genomes. The transfer, replication, and adaptive modules in pAbe229-15, and the stability module in pAbe229-9, were bordered by sites potentially recognized by XerC/XerD site-specific tyrosine recombinases, thus suggesting a potential mechanism for their acquisition. The presence of Rep_3 and ColE1-based replication modules, different *mob* genes, distinct adaptive functions including resistance to heavy metal and other environmental stressors, as well as antimicrobial resistance genes, and a high content of XerC/XerD sites among HPC229 plasmids provide evidence of substantial links with bacterial species derived from both environmental and clinical habitats.

## Introduction

Plasmids are extra-chromosomal self-replicating DNA molecules that act as efficient vectors of horizontal gene transfer (HGT) across bacterial populations, thus facilitating adaptation of particular individuals and derived clonal lineages to novel and/or fluctuating environmental conditions [1,2]. Bacterial plasmids are assemblies of different modules encompassing replication, mobilization and stability functions in what is defined as the plasmid backbone [3]. This conserved structure is generally accompanied by accessory genes that provide adaptive functions, including pathogenicity/virulence, antimicrobial resistance, and/or other traits depending on the selective context [1,3]. Plasmids are also carriers of other mobile genetic elements such as insertion sequences (IS), transposons, and different genomic islands, thus becoming vectors for the transport of information from a communal gene pool [1]. They additionally provide scaffolds where different genetic rearrangements can occur via different homologous and non-homologous recombination processes, events that contribute not only to their own evolution but also the survival of their hosts under different evolutionary pressures [4].

The identification of plasmid types provides relevant information about their impact on the physiology of their hosts and their modes of transmission [3]. Current plasmid typing schemes exploit the more conserved backbone modules associated with replication (Rep typing) and/or mobility (MOB typing) [3,5]. Still, a caveat of these schemes is that plasmids lacking Rep or MOB genes escape classification schemes [6]. In this context, the rapid advance of whole genome sequence methodologies (WGS), by providing complete (or near-complete) plasmid sequences, has helped to characterize not only of the plasmid backbone but also of accessory genes including the complete repertoire of mobile genetic elements, and the identification of potential recombinatorial hot spots in the plasmid sequence. This may provide many clues on the evolutionary processes that shaped their structures and drove their persistence in a given bacterial group, as well as their potentiality of dissemination to novel hosts [7–12].

We have recently demonstrated [13] that *A*. *baumannii* plasmids can exploit the XerC/XerD site-specific recombination system [14] to mediate fusions and resolutions of different replicons, some of them carrying *bla*_OXA-58_ and *aphA6* genes conferring carbapenem and aminoglycoside resistance, respectively. This may represent a powerful mechanism not only to drive plasmid structural rearrangements but also host range expansions [13]. Several authors have proposed in fact that the *Acinetobacter* XerC/XerD recombination system may mediate the mobilization of different DNA segments carrying adaptive, that includes antimicrobial resistance, and heavy metal resistance, or stability traits such as toxin-antitoxin (TA) genes conforming part of the plasmid backbone [6,13,15–18].

We have recently characterized by WGS a carbapenem-resistant *Acinetobacter bereziniae* strain isolated in Argentina designated HPC229 [19]. HPC229 carries a *bla*_NDM-1_-bearing carbapenem-resistance plasmid of 44 kbp designated pNDM229 [20], which displays a similar structure to plasmid pNDM-BJ01 carried by an *A*. *lwoffii* strain isolated in China [21]. This strongly suggested that pNDM229 was acquired by HPC229 as the result of HGT from other member of the genus, and selected due to the antimicrobial pressure common to the clinical setting [20,22]. As a species, *A*. *bereziniae* has a wide distribution and has been isolated from various sources including soils, vegetables, animals, and human-associated environments such as waste waters [23–26]. More recently, *A*. *bereziniae* has been also increasingly associated to healthcare-associated infections in humans [25,27]. In fact, although *A*. *bereziniae* isolates were in the past generally susceptible to most antimicrobials of clinical use [24], since 2010 a number of carbapenem-resistant clinical strains bearing IMP-, SIM-, VIM- or NDM-type metallo-β-lactamases (MβL) have been reported [20,22,25,28]. MβL genes are generally carried in these strains by plasmids, suggesting both the mechanism of their acquisition and potential spread to other Gram-negative species [20,25]. Contrary to the acquired resistance plasmids mentioned above, the available information on the natural plasmids circulating among the population of this species and which may also contribute to the adaptation to different habitats is rather limited. This general lack of information on the *A*. *bereziniae* intrinsic plasmid repertoire reflects the case of most other species of the genus except for *A*. *baumannii* [29,30].

Here, we characterized in detail the whole plasmid content of the HPC229 strain, in an attempt to gain information on the intrinsic repertoire of *A*. *bereziniae* plasmids lacking antimicrobial resistance determinants but putatively contributing to other traits such as bacterial persistance. This included a comprehensive sequence analysis not only of the modules related to replication, stability, and mobilization, but also the identification of mobile elements, adaptive genes, and XerC/D recognition sites which may drive structural plasticity in their sequences and thus help in their persistence and the adaptation of their host(s) to different environments.

Parts of the results of this work were presented in the Symposium of Plasmid Biology 2018, Seattle, USA (Abstract 101).

## Materials and methods

### Strain assignation based on ANI calculations

Average nucleotide identity values (ANI) [31] were calculated and used for *Acinetobacter* species assignation. ANI were calculated from the published draft genome sequence data of the strains under study using orthoANI [32]. Briefly, each genome pair consisting in a query genome *versus* the reference strain CIP70.12 genome were first split into 1,020 bp fragments, which were used to run bidirectional BlastN searches (cut-off value higher than 70%). Only orthologous fragment pairs satisfying this requirement [32] were then taken into consideration for calculating ANI. An ANI cut-off value higher than 95% was adopted to define a given *Acinetobacter* species [33]. The accuracy of the species assignment by this procedure was evaluated by calculating the ANI corresponding to the non-*A*. *bereziniae* strains *A*. *guillouiae* CIP 63.46 and *A*. *gerneri* DSM 14967, which represent two *Acinetobacter* species showing the closest phylogenetic affiliation to *A*. *bereziniae* [23, 26].

### HPC229 plasmids assembly and annotation

*Acinetobacter bereziniae* HPC229 is a carbapenem resistance clinical strain isolated from a blood sample of a 53-year-old female patient with leukemia [20]. The draft genome sequence of this strain was obtained using a 454 pyrosequencing platform (Roche Diagnostics) [19]. The revised assembled genome has been deposited at DDBJ/ENA/GenBank under the accession LKDJ00000000.2. Newly assembled genome sequences were annotated using the pipeline available at National Center for Biotechnology Information (NCBI).

Suspected plasmid sequences were analysed and assembled into putative replicons *in silico*, and the gaps inferred in the sequences were closed by PCR conducted on plasmid extracts with the aid of specifically designed primer pairs (S1 Table). The DNA sequences of all amplicons obtained in these assays were verified at the Sequencing Facility of Maine University. The overall analyses indicated the presence, besides of pNDM229, of five additional plasmids lacking antimicrobial resistance determinants (Table 1). The ORFs predicted by the NCBI pipeline in these plasmids were compared with the protein sequences deposited in the GenBank database using BlastP [34]. The CGview tool with default parameters (http://cgview.ca/) [35] was used for the visualization of GC content and GC skew.

**Table 1:** Characteristics of *A*. *bereziniae* HPC229 plasmids.

Plasmid	Size (kbp)	RepB (replication initiator protein) assignment ^a^	Iteron sequences (length)^b^	No. of direct repeats	TA systems	MOB-subfamily classification^b^	Resistance determinants^c^
pAbe229-114	114	pXBB1-8 (99%)/AR3G4	5´-AAGTGGAAGGCCTGTCACTAAT-3´ (22 bp)*	6	*hipA/B*, *fic/yhfG*, *higA/B*, *higA2/B2*, *hicB*	MOB_P111_	*cadAR* and *cadAR*-like, *nrpAB*, *acr3*, *cusABCF*
pNDM229	44.5	-	-	-	*zeta* toxin	MOB_Q1_	*bla*_NDM-1_, *aphA6*, *ble*_MBL_
pAbe229-15	15.3	pMMD (100%)/AR3G3	5´-TAACTATGACGGATTGACTAC-3´ (21 bp)**	4	*higA2/B2*	MOB_Q1_	-
pAbe229-9	9.1	pAV1 (85%)/AR3G6	5´-ACCTATACCACACCAAAAAGTC-3´ (22bp)**	4	*splT/A*, *doc*	MOB_Q1_	-
pAbe229-4	4.4	-	-	-	*relE/B*	-	-
pAbe229-1	1.3	-	-	-	-	-	-

^a^According to the classification of *Acinetobacter* Rep_3 superfamily proteins [30]. The percentages of identity to representative members of a different subgroups (AR3G) are indicated between brackets.

^b^Iteron sequences were identified using the Tandem Repeats Finder program available at (https://tandem.bu.edu/trf/trf.html) and manually curated. An asterisk (*) indicates the presence of imperfect repeat sequences (the consensus pattern was calculated by Tandem Repeats Finder). Two asterists (**) indicate four perfect repeats.

^c^*cadAR* and *cadAR*-like operons encoding putative Co/Zn/Cd ions resistance systems; *nrpAB* operon encoding putative a Ni ion resistance system; *acr3*, putative arseniate resistance; *cusABCF* operon encoding putative Cu ion resistance system.

### Plasmid isolation and S1 analysis

HPC229 plasmids were extracted using the Wizard DNA purification kit (Promega, Madison, WI, USA), and analyzed by 0.7% agarose gel electrophoresis and ethidium bromide staining following described procedures [13]. S1 nuclease treatment of plasmid extracts (S1 Fig) was conducted essentially as described previously [13].

### Comparative sequence analyses

The presence of mobile genetic elements among HPC229 plasmids was inspected using BlastN [34]. Only hits equal or higher than 70% nucleotide identity and minimum alignment lengths of 1,000 bp for pAbe229-114 or 300 bp for pAbe229-15, pAbe229-9 and pAbe229-4 were considered for further analysis. Graphical displays of nucleotide homologies between HPC229 plasmids with other *A*. *bereziniae* genomes were obtained using CGview (http://cgview.ca/) [35] employing a BlastN-homology search with an E-value cut-off of 1e-15.

### Bioinformatic identification of diverse genetic elements

Classification of membrane transport proteins was done using the Transporter Classification Database program (http://www.tcdb.org/) [36]. The screening for type II TA systems was conducted using TADB (http://bioinfo-mml.sjtu.edu.cn/TADB/) [37] and RASTA-Bacteria (http://genoweb1.irisa.fr/duals/RASTA-Bacteria/) [38] web-based search tools. Data retrieved from these databases were manually curated and complemented with BlastP-homology searches against the NCBI Protein database [34].

Insertion sequences (IS) were detected using IS Finder (https://www-is.biotoul.fr/) [39] and ISSaga [40]. The designation of novel IS elements were provided by the curators of the IS database [39], while transposon (Tn) designations were assigned by the Tn Number Registry [41]. The Tandem Repeats Finder program [42] was used to identify direct repeats by using default parameters. The GenSkew program (http://genskew.csb.univie.ac.at/) was used to predict global minima skew values generally associated to plasmid origins of replication [43].

### *A*. *bereziniae* plasmid classification based on the comparison of Rep proteins

*A*. *bereziniae* plasmids bearing *rep* replication initiator protein (Rep) genes were classified following the scheme recently proposed by Salto et al. [30] for *Acinetobacter* plasmids. First, a search for known plasmid Rep domains as judged by the NCBI conserved domain database [44] was conducted among the *A*. *bereziniae* plasmid sequences reported in this work and those retrieved from *A*. *bereziniae* genomes available in the GenBank public database. Putative Rep candidates were then used as queries for a BlastP search against a local protein database that included all the Rep sequences reported by Salto et al. [30], and subsequently assigned to the corresponding *Acinetobacter* AR3G groups following the criterium described by these authors.

### Classification of *A*. *bereziniae* plasmids encoding MOB proteins

*A*. *bereziniae* plasmids bearing relaxase genes were classified following the scheme of MOB classification in families and subfamilies essentially following described procedures [30]. First, a local database that included all the MOB proteins used for the classification of *Acinetobacter* relaxases into MOB groups and subgroups was constructed [30]. Then, a search for proteins encoded in *A*. *bereziniae* genomic sequences available in the GenBank database with conjugation and/or mobilization functions was performed. This search retrieved 12 predicted protein sequences encoding for putative relaxases as judged by their homologies to described relaxase domains (NCBI conserved domain database) [44]. Among these 12 sequences, eleven showed the pfam03389 MobA/MobL domain and the other (encoded in pAbe229-114 and designated TraI*) showed the pfam03432 Relaxase/Mobilization nuclease domain. The eleven proteins bearing the pfam03389 domain were subjected to further phlylogenetic analysis. For this purpose, their N-terminal domains (first 300 amino acid residues) were first aligned with the MOB proteins of local database (see above) using MEGA6.06 [45] by employing ClustalW [46] with default parameters, and a Maximum-Likelihood (ML) phylogenetic tree was then generated using these alignments. To determine the best-fit protein substitution model, the tool included in MEGA6.06 was employed. This resulted in the use of the LG+G+I substitution model, taking into account the Akaike information criterion (AIC). Bootstrap values (100 replications) were also calculated using MEGA6.06 [45].

In the case of the TraI* relaxase encoded in pAbe229-114 (see above), the assignment was done using the best-hit match in a BlastP search against the relaxase local database.

### Testing the functionality of the tranfer regions present in *A*. *bereziniae* plasmids

Potentially mobilizable plasmids possessing a predicted origin of transfer and *mob* genes codifying relaxase proteins, necessarily requiere other conjugative functions provided *in trans* to allow transfer to a new host [30,47]. To evaluate the ability of *A*. *bereziniae* plasmids encoding predicted *mob* genes to undergo mobilization, we essentially followed previously described procedures by other authors [30,47]. In the case of pAbe229-114, a 2,682 bp fragment encompassing *oriT*, *traJ* and Δ*traI* genes (see below) was amplified by PCR with primers mob114-F and mob114-R (S1 Table), using HPC229 genomic DNA as template. This amplicon was then cloned into pGEM-T-Easy (Promega, Madison, WI, USA), thus generating pGEM-mob114 following conventional *E*. *coli* DH5α transformation procedures. pGEM-mob114 was then used to transform *E*. *coli* Eco S17 [48] harboring the conjugative plasmid pRP4 integrated into the chromosome. The conjugative transfer of pGEM-mob114 from Eco S17 was then tested in a mating assay using rifampicin resistant (rif^R^) *E*. *coli* DH5α as acceptor [20]. In the cases of pAbe229-15 and pAbe229-9, 2,803 bp and 2,562 bp fragments of the corresponding transfer regions encompassing orf15, *oriT*, and *mobA* gene (see below) were amplified by PCR with mob15-F and mob15-R for pAbe229-15, or mob9-F and mob9-R primers for pAbe229-9 (S1 Table) using HPC229 genomic DNA as template. These amplicons were cloned into pGEM-T-Easy (Promega, Madison, WI, USA), and the transferability of the obtained plasmids was tested as described above. The transconjugants in each case were selected on LB agar plates containing 100 μg/ml ampicillin and 150 μg/ml rifampicin, and resistant colonies were counted after an overnight incubation at 37°C. The presence on selected transconjugants of the transfer regions from the tested plasmids was evaluated by PCR with the corresponding primers (S1 Table). Conjugation frequencies were indicated as the average number of transconjugants (in UFC/ml) per donor cell obtained in three independent experiments.

### Search for XerC/D recombinases recognition sites in *A*. *bereziniae* plasmids

HPC229 plasmid sequences were first queried using Fuzznuc (http://www.bioinformatics.nl/cgi-bin/emboss/fuzznuc) with default parameters with an ambiguous XerC/D nucleotide recognition sequence (NNTNYKYATAANNNNYWTTATSTKAWNN, whereY = C/T, K = G/T, W = A/T, S = G/C, N = A/T/C/G), inferred from the consensus 28-mer XerC/D recognition sequence determined for *A*. *baumannii* plasmids [13]. This search found 9 XerC/D recognition sites among these plasmids, which were complemented with 3 additional sites detected after a thoughtful visual inspection of plasmid sequences.

XerC/D recognition sites bearing a 6 nt-central region (cr6-XerC/D) detected in HPC229 plasmids were then used to infer a (degenerate) consensus XerC/D recognition sequence: (DHWYCKHATAANNNNNNTTATGTTAADT; where D = A/G/T, H = A/C/T), and used as query to identify equivalent sites in *A*. *bereziniae* sequences. A logo depicting the *A*. *bereziniae* XerC/D site frequency plot was then constructed using this information with the tool available at https://weblogo.berkeley.edu/logo.cgi.

### Ethics statement

This study involved sequence analysis of *A*. *bereziniae* plasmids and did not implicate human specimens or participants.

## Results and discussion

### Analysis of plasmids harbored by *A*. *bereziniae* HPC229

**Fig 1:**
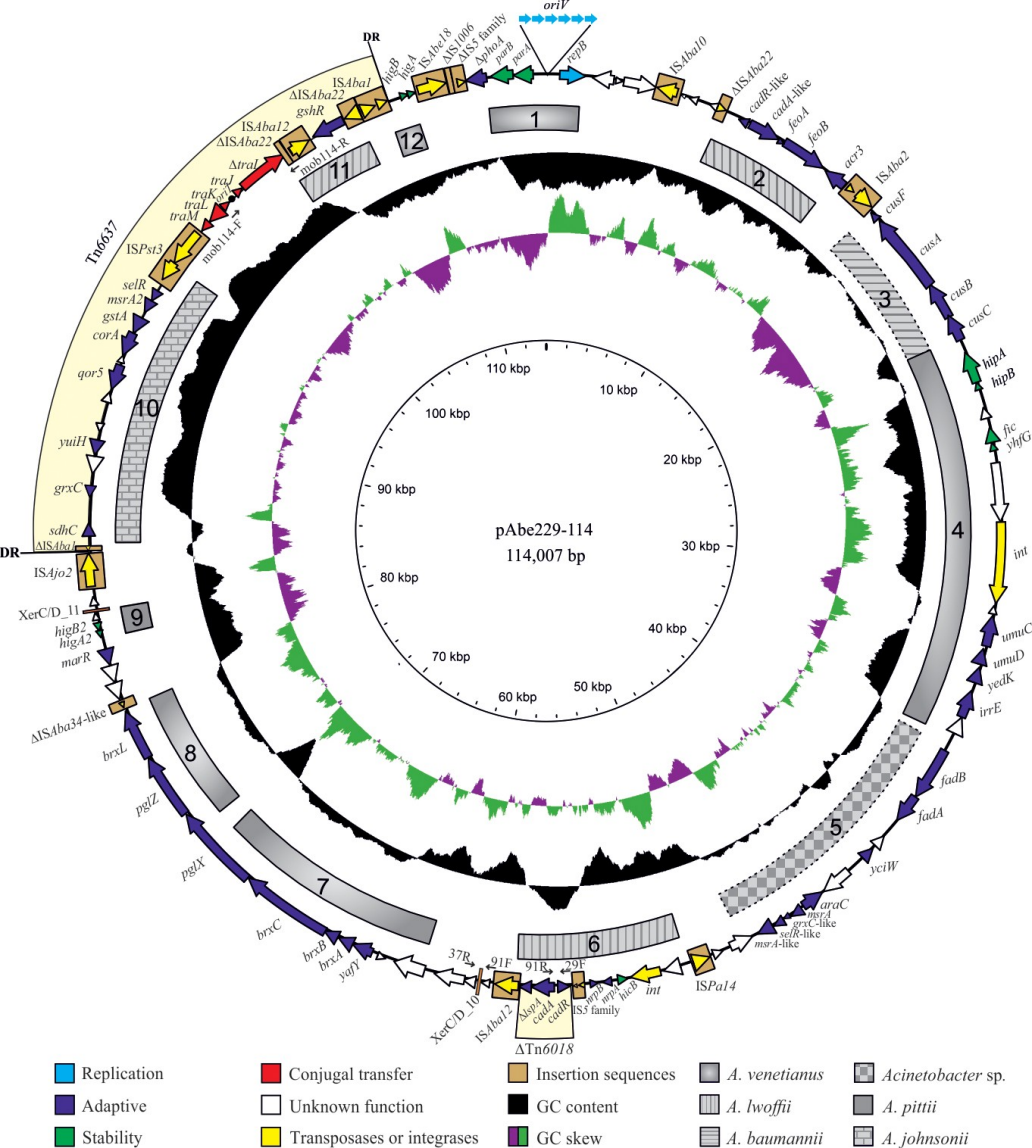
Schematic representation of pAbe229-114 plasmid. ORFs are shown as arrows indicating the direction of transcription. Disrupted/incomplete genes are indicated with a “Δ” symbol preceding the gene denomination. The six consecutive blue light arrows upstream of the *repB* gene denote the predicted iteron sequences linked to the *oriV* region (see Table 1 for details). The putative *oriT* located within the conjugal/transfer region is indicated with a closed circle. The Tn*6637* transposon bracketed by IS*Aba1* elements (one of them truncated, ΔIS*Aba1*) is highlighted in light yellow. The 9-bp (AATAAAGAT) direct repeats found at the insertion target site (DR) are indicated next to the external inverted repeats. From the outer circle inward, the circles display: i) the predicted ORFs. The colored arrows describe the location, identification, and direction of transcription of genes with described functions in databases. ORFs with undescribed functions are indicated by open arrows, ii) in grey with different designs, homologous regions described in plasmids/chromosomes of *Acinetobacter* non-*bereziniae* strains (see Table 2 for details), iii) GC content relative to the mean GC content of the plasmid, iv) GC skew, where green and purple represent positive and negative skew, respectively, v) scale in kbp. The hybridation sites of the PCR primer pairs used to verify the structure of pAbe229-114 as well as those used to amplify the transfer region (see Materials and Methods) are also indicated (S1 Table).

**Fig 2:**
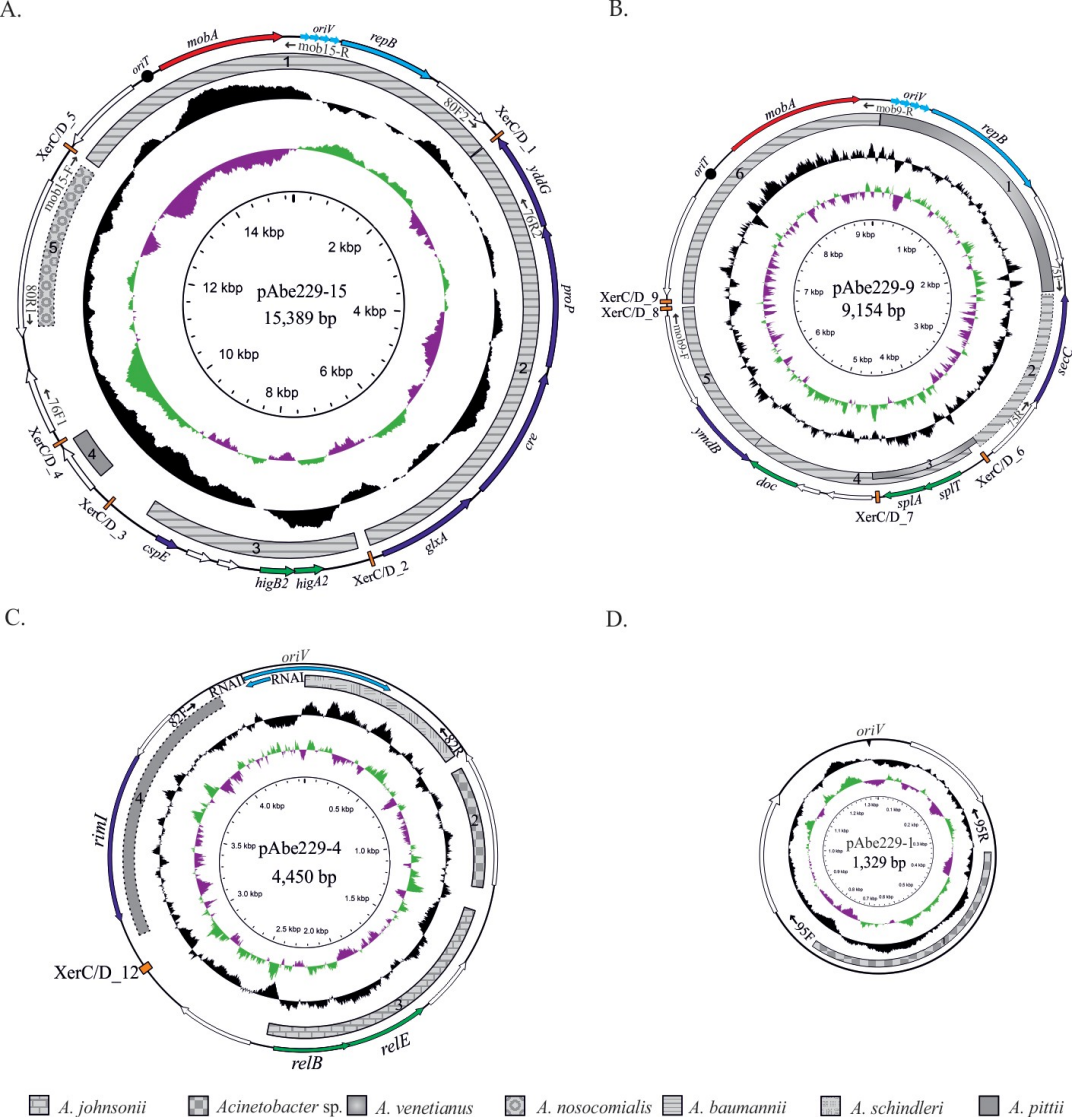
**Schematic representation of plasmids pAbe229-15 (A), pAbe229-9 (B), pAbe229-4 (C) and pAbe229-1 (D).** The regions encoding RNAI- and RNAII-homologous sequences in pAbe229-4 are highlighted in light blue, and the direction of transcription is also indicated in each case. In pAbe229-1, the dark triangle denotes a high-AT region (putative *oriV*) predicted on the basis of the cumulative GCskew (see the text). Plasmids are not drawn to scale. For details, see the legend to Fig 1.

The different contigs obtained after HPC229 genomic pyrosequencing were extensively analyzed to search for plasmid sequences other than pNDM229 characterized in detail previously [20]. Among them we identified 5 putative plasmid sequences, which were thoughtfully assembled *in silico* and the resulting inferred final structures subsequently validated by PCR using specifically designed primer pairs (S1 Table, Figs 1 and 2). Therefore HPC229 harbors, besides pNDM229, five other plasmids of approximate sizes of 114, 15.4, 9.1, 4.4, and 1.3 kbp which will be hereafter designated pAbe229-114, pAbe229-15, pAbe229-9, pAbe229-4, and pAbe229-1, respectively (Table 1; GenBank accession numbers are provided in S1 Table). Conventional plasmid extraction combined with S1 nuclease treatment and agarose gel electrophoresis analysis of the digested material (S1 Fig) indicated the presence of four DNA bands of approximate sizes of 15, 9, 4 and 1.3 kbp, additionally supporting the presence of pAbe229-15, pAbe229-9, pAbe229-4, and pAbe229-1 in these cells. The larger plasmids were not visualized by this procedure, a situation thay may have resulted from their much larger sizes and/or copy number.

**Table 2:** Homologous regions in *Acinetobacter* spp.

***Acinetobacter* non-*bereziniae* strains**								
**HPC229 plasmids**	**Strain, plasmid or chromosome (chr)**	**Position in plasmid (bp)**	**Length (bp)**	**% identity**	**Accession number**	**Region**	**Functional** **regions**^**a**^	
pAbe229-114	*A*. *venetianus* VE-C3, pAV3	111,696–1,554	3,868	99	NZ_ALIG01000010.1	1	R and S	
	*A*. *lwoffii* ZS207, pmZS	7,297–12,565	5,268	95	NZ_CP019144.1	2	A	
	*A*. *baumannii* LAC-4, chr	14,413–20,503	6,091	95	NZ_CP007712.1	3	A	
	*A*. *venetianus* VE-C3, pAV3	20,504–36,960	16,457	99	NZ_ALIG01000010.1	4	S and A	
	*Acinetobacter* sp. WCHA45, chr	37,327–48,798	11,472	99	NZ_CP028561.1	5	A	
	*A*. *lwoffii* ED9-5a, pALWED3.1	51,114–58,084	6,971	99	KX528687.1	6	S and A	
		58,075–59,688	1,614	98				
	*A*. *venetianus* VE-C3, pAV3	61,877–71,694	9,818	95	NZ_ALIG01000010.1	7	A	
	*A*. *venetianus* VE-C3, pAV3	72,546–78,258	5,713	92		8	A	
	*A*. *pittii* AP_882, pOXA58-AP_882	81,384–82,478	1,097	98	CP014479.1	9	S	
	*A*. *johnsonii* XBB1, pXBB1-9	85,054–96,889	11,836	99	NZ_CP010351.1	10	A	
	*A*. *lwoffii* ED23-35, pALWED1.1	102,998–106,433	3,436	99	KX426227.1	11	A	
	*A*. *venetianus* VE-C3, pAV3	106,430–108,753	1,140	99	NZ_ALIG01000010.1	12	S	
pAbe229-15	*A*. *baumannii*, pMMD	13,048–2,229	4,545	98	GQ904226.1	1	R and T	
	*A*. *baumannii* ABNIH28, pABA-2f10	2,176–6,882	4,706	99	NZ_CP026129.1	2	A	
	*A*. *baumannii* AR_0052, unnamed4 plasmid	7,000–9,169	2,169	92	CP027186.1	3	S and A	
	*A*. *pittii* WCHAP005046, pOXA58_005046	9,748–10,203	455	96	NZ_CP028573.1	4	U	
	*A*. *nosocomialis* 6411, chr	11,351–13,008	1,657	78	NZ_CP010368.1	5	U	
pAbe229-9s	*A*. *venetianus* VE-C3, pAV1	1–2,132	2,132	79	DQ278485.1	1	R	
	*A*. *baumannii* ABNIH28, chr	2,252–3,632	1,380	82	NZ_CP026125.1	2	A	
	*A*. *venetianus* VE-C3, pAV1	3,621–4,516	895	88	NC_010309.1	3	S	
	*A*. *baumannii* D36, pD36-4	3,712–5,541	1,829	99	NZ_CP012956.1	4	S	
	*A*. *baumannii* D36, pD36-4	5,533–6,799	1,266	81		5		
	*A*. *baumannii*, pMMD	6,830–313	2,637	94	GQ904226.1	6	T	
pAbe229-4	*A*. *schindleri* ACE, p3AsACE	1–692	691	70	NZ_CP015618.1	1	U	
	*Acinetobacter* sp. ACNIH2, pACI-235c	745–1,227	482	82	NZ_CP026414.1	2	U	
	*A*. *johnsonii* XBB1, pXBB1-4	1,246–2,378	1,132	87	NZ_CP010355.1	3	S	
	*A*. *pittii* YMC2010/8/T346, chr	3,034–4,104	1,070	70	NZ_CP017938.1	4	A	
pAbe229-1	*Acinetobacter* sp., pM131-11	330–795	465	87	NC_025117.1	1	U	
***A*. *bereziniae* strains**								
**HPC229 plasmids**	**Strain**^**b**^	**Position in plasmid (bp)**	**Length (bp)**^**c**^	**% identity**	**Accession number**	**Region**^**d**^	**Functional** **regions**	**Total cover (bp)**
pAbe229-114	*A*. *bereziniae* NIPH3	20,504–36,960	16,456	99	APPK01000003.1	Abe4	S and A	47,779
		37,431–40,931	3,500	86	APPK01000060.1	Abe5*	A	
		51,114–53,896	2,782	97	APPK01000003.1	Abe6*	A	
		54,413–71,707	17,593	98	APPK01000004.1	Abe6*; Abe6-7; Abe7*	A	
		73,566–81,014	7,448	99	APPK01000004.1	Abe8; Abe8-10	A	
	*A*. *bereziniae* CHI-40-1	20,504–36,960	16,456	94	CDEL01000167.1	Abe4	S and A	42,386
		37,431–40,929	3,498	86	CDEL01000296.1	Abe5*	A	
		51,114–53,998	2,884	99	CDEL01000167.1	Abe6*	A	
		53,998–58,165	4,167	98	CDEL01000167.1	Abe6*	S and A	
		59,646–61,052	1,406	97	CDEL01000305.1	Abe6*; Abe6-7	A	
		61,089–63,912	2,823	99	CDEL01000063.1	Abe6-7; abe7*	A	
		63,854–71,428	7,574	92	CDEL01000026.1	Abe7*	A	
		72,538–74,328	1,790	90	CDEL01000300.1	Abe8*	A	
		76,598–78,386	1,788	92	CDEL01000225.1	Abe8*	A	
	*A*. *bereziniae* WC-743	8,638–11,719	3,081	77	AMFQ01000090.1	Abe2*	A	33,324
		20,504–23,881	3,377	98	AMFQ01000055.1	Abe4*	S	
		23,875–34,350	10,475	98	AMFQ01000007.1	Abe4*	S and A	
		37,431–40,929	3,498	86	AMFQ01000023.1	Abe5*	A	
		52,562–61,354	8,792	97	AMFQ01000126.1	Abe6*; Abe6-7; Abe7*	S and A	
		61,348–65,449	4,101	99	AMFQ01000094.1	Abe7*	A	
	*A*. *bereziniae* CIP 70.12	7,847–9,642	1,795	99	APQG01000008.1	Abe2*	A	30,050
		36,962–50,325	13,363	99	APQG01000004.1	Abe5	A	
		84,864–96,322	11,458	96	APQG01000007.1	Abe10*	A	
		102,999–106,433	3,434	99	APQG01000007.1	Abe11	A	
	*A*. *bereziniae* 507_ABAU	8,107–11,787	3,680	94	JVEK01000235.1	Abe2*	A	26,037
		23,104–36,960	13,856	97	JVEK01000235.1	Abe4*	S and A	
		37,431–40,929	3,494	86	JVEK01000265.1	Abe5*	A	
		51,114–56,121	5,007	99	JVEK01000235.1	Abe6*	S and A	
	*A*. *bereziniae* KCTC23199	7,847–9,641	1,794	99	BBLJ01000102.1	Abe2*	A	23,722
		36,962–50,325	13,363	99	BBLJ01000069.1	Abe5	A	
		84,864–86,792	1,928	99	BBLJ01000081.1	Abe10*	A	
		91,925–96,322	4,397	96	BBLJ01000078.1	Abe10*	A	
		104,193–106,433	2,240	99	BBLJ01000078.1	Abe11	A	
	*A*. *bereziniae* Ag2	34,737–36,634	1,897	86	LBNA01000036.1	Abe4*	A	5,430
		37,431–40,964	3,533	86	LBNA01000006.1	Abe5*	A	
pAbe229-15	*A*. *bereziniae* WC-743	2,176–6,882	4,706	97	AMFQ01000126	Abe2	A	4,706
	*A*. *bereziniae* 507_ABAU	107–1,383	1,276	81	JVEK01000173.1	Abe1*	R	3,556
		1,900–2204	304	74	JVEK01000173.1	Abe1*	A	
		13,128–15,104	1,976	76	JVEK01000173.1	Abe1*	T	
	*A*. *bereziniae* CHI-40-1	1,458–2,107	649	71	CDEL01000197.1	Abe1*	A	2,571
		6,904–7,384	480	87	CDEL01000201.1	Abe2-3; Abe3*	A	
		7,361–8,047	686	82	CDEL01000088.1	Abe3*	S	
		9,479–10,235	756	98	CDEL01000195.1	Abe3-4; Abe4	U	
	*A*. *bereziniae* CIP 70.12	8,157–9,170	1,013	78	APQG01000012.1	Abe3*	A	1,013
	*A*. *bereziniae* KCTC23199	8,157–9,171	1,013	78	BBLJ01000051.1	Abe3*	A	1,013
pAbe229-9	*A*. *bereziniae* 507_ABAU	3,760–4,445	685	84	JVEK01000068.1	Abe3*	S	2,652
		6,902–8,869	1,967	75	JVEK01000173.1	Abe6*	T	
	*A*. *bereziniae* CHI-40-1	1,539–2,170	631	77	CDEL01000197.1	Abe1*	U	1,422
		3,713–4,504	791	91	CDEL01000314.1	Abe3*	S	
	*A*. *bereziniae* WC-743	3,760–4,445	685	84	AMFQ01000013.1	Abe3*	S	685
	*A*. *bereziniae* NIPH3	3,780–4,445	665	84	APPK01000024.1	Abe3*	S	665
	*A*. *bereziniae* CIP 70.12	1–315	315	71	APQG01000002.1	Abe1*; Abe6*	U	315
	*A*. *bereziniae* KCTC23199	1–315	315	71	BBLJ01000084.1	Abe1*; Abe6*	U	315
pAbe229-4	*A*. *bereziniae* Ag2	31–2,383	2,352	90	LBNA01000007.1	Abe1; Abe1-2; Abe2; Abe2-3; Abe3	S	2,352
	*A*. *bereziniae* CHI-40-1	248–692	444	74	CDEL01000176.1	Abe1*	U	444

^a^Abbreviations: Replication: R; Stability: S; Adaptive: A; Transfer: T; and unknow: U.

^b^*A*. *bereziniae* XH901 genomic data (GenBank accession number CP018259.1) was not included in this analysis since no plasmid sequences are available for this strain in databases.

^c^Hits with high similarity, at least 70% nucleotide identity, and a minimum alignment length of 1000 bp (for the case of pAbe229-114) or 300 bp (for pAbe229-15, pAbe229-9, pAbe229-4) are shown. These parameters were arbitrary chosen.

^d^A segment located between two given Abe regions is indicated by the flanking regions separated with a hyphen (*e*.*g*. Abe1-2 is the segment between Abe1 and Abe2). Abe regions with asterisks (*) imply that the query coverage is partial.

### pAbe229-114 encodes five heavy metal resistance operons and a phage resistance system

pAbe229-114, the largest plasmid found in these cells, contains 114,007 bp, displays a GC content of 42.1% (Fig 1), and contains 111 ORFs from which 84 (i. e., 76%) encode proteins exhibiting known functions (S2 Table). It shows an *oriV-repB* replication region, with an encoded RepB replication initiator protein of the Rep_3 superfamily (pfam01051 conserved domain) displaying complete amino acid identity with their homologs encoded in *A*. *ursingii* UMB1319 and in *A*. *nosocomialis* 2010S01-197 (WP_043972782.1; S2 Table). pAbe229-114 was assigned to the *Acinetobacter* AR3G4 plasmid group, following the classification of *Acinetobacter* plasmids based on Rep_3 proteins recently proposed [30] (S3 Table). The *oriV* region, which is located 549 bp upstream of the *repB* start codon, contains six imperfect direct repeats of 22 bp each (Table 1, Fig 1) likely corresponding to iteron sequences playing roles during the initiation of replication and plasmid copy number control [49]. Comparative sequence analyses of the whole replication module including *repB* and the iterons indicated 99% nucleotide identity with a similar region located in plasmid pXBB1-8 of *A*. *johnsonii* XBB1 recovered from hospital sewage (Table 1) [50].

In addition to the replicative module, pAbe229-114 encodes different stability functions, including ParA/ParB as well as HipA/HipB, Fic/YhfG, HigA2/HigB2, and HigA/HigB TA systems. For each of these TA systems, homologous were detected in other *Acinetobacter* species (Fig 1, S2 Table).

pAbe229-114 also contains a conjugative/transfer region (*traMLKJ*-Δ*traI*; S2 Table) in which the relaxase gene (pfam03432) is partially deleted at the 3’ region (hence the Δ*traI* designation). The translated protein (TraI*), however, still retains a complete relaxase domain. The conjugative/transfer region mentioned above showed 91% nucleotide identity with a homologous segment displaying a complete *traI* gene found in the conjugative plasmid pQKH54 (AM157767.1) from an uncharacterized bacterium [51]. A BlastP search using as query the N-terminal first 300 amino acids (relaxase domain) of TraI* showed 92% identity with a homologous relaxase corresponding to MOB_P111_ subfamily (Table 1) [30].

Comparative nucleotide sequence analysis of pAbe229-114 against *Acinetobacter* genomes deposited in databases allowed us to detect homology between several regions of this plasmid with other sequences present in other clinical and environmental species of the genus (Table 2; see the upper part of the Table for sequences present in *Acinetobacter* non*-bereziniae* genomes). For instance, replication and stability functions (region 1, Fig 1, and Table 2) showed homology with a region carried by pAV3 from the environmental *A*. *venetianus* VE-C3 strain [52]. In addition, three of the pAbe229-114 TA systems, namely HipA/HipB, Fic/YhfG and HigA/HigB, were also identified in pAV3 (regions 4 and 12, respectively, Fig 1, and Table 2). Their presence likely fulfills an important role for plasmid stability in both replicons [53]. In turn, the HigA2/HigB2 TA system found in pAbe229-114 (region 9, Fig 1, and Table 2) has probably been independently acquired from other sources, as inferred from the high percentage of identity between this region and a homologous sequence identified in *A*. *pittii*.

The region of pAbe229-114 encoding heavy metal ions resistance functions shows homology with a region located in plasmid pmZS of the *A*. *lwoffii* ZS207 environmental strain (region 2, Fig 1, and Table 2). Moreover, the 6,091 bp-segment encoding a RND efflux pump related to copper ion resistance showed homology with a region (region 3, Fig 1, and Table 2) detected inside the GI2-genomic island located on the chromosome of the *A*. *baumannii* LAC-4 clinical strain [54]. In turn, a 6,971 bp-DNA segment encoding cobalt-zinc-cadmium ions resistance contained in a truncated Tn*6018* element, and the *nrpAB* genes encoding nickel ion resistance [55,56], exhibited homology with pALWED3.1 of the *A*. *lwoffii* ED9-5a environmental strain (region 6, Fig 1, and Table 2). Other adaptive regions probably involved in oxidative stress resistance encode functions responsible for the reduction of methionine sulfoxides in damaged proteins (regions 5 and 10, Fig 1, and Table 2). These regions show homology to DNA sectors detected in *Acinetobacter* sp. WCHA45 and *A*. *johnsonii* plasmid pXBB1-9, respectively. The *fabAB* genes, which are located within region 5, have been related to intrinsic resistance to aminoglycosides [57,58]. Furthermore, a locus encoding a BREX system (S2 Table) reported to be involved in defense against bacteriophages [59], displayed homology with a cluster present in plasmid pAV3 from *A*. *venetianus* VE-C3 (regions 7 and 8, Fig 1, and Table 2). Remarkably, a region of this locus of around 1 kbp encompassing the central part of *pglX* (Table 2) is not conserved between these plasmids. *pglX* encodes a protein containing an adenine-specific DNA methyltransferase motif, and variability in this gene has been documented and linked to a possible phase variation playing regulatory functions [59].

Of note, the overall observations above uncovered extensive regions of identity between the plasmid backbones of *A*. *bereziniae* pAbe229-114 and *A*. *venetianus* VE-C3 pAV3 (Fig 1, Table 2). Comparative nucleotide sequence analyses between these two plasmids indicated in fact that they share around 50% equivalent sequences, including the replication and stability regions and part of the adaptive region. The additional presence of an integrase, two TA systems, as well as UmuC and UmuD error-prone DNA polymerase V subunit genes [60] in this region led us to conclude that this region constitutes a genomic island probably acquired by HGT. Besides the similarities between pAbe229-114 and other plasmids summarized above, unique regions were also identified in this plasmid including the conjugal transfer region as well as some segments involving genes contiguous to IS elements corresponding to the adaptive regions (Fig 1, S2 Table).

A search for mobile genetic elements in pAbe229-114 indicated the existence of 15 different IS (including complete IS and IS remnants) assigned to 7 different families (Table 3). The presence among them of IS*Aba12* and IS*Aba22* in multiple copies is consistent with previous observations indicating the ubiquitous distribution of these two IS among *Acinetobacter* genomes [61]. This suggested that pAbe229-114 has undergone several structural rearrangements, some resulting from IS insertions. Most of the IS shown in Table 3 have been reported previously in different members of the *Acinetobacter* genus [61]. However, we identified a novel IS element, which received the designation IS*Abe18* by the ISSaga database [36]. This IS*Abe18* is flanked by 9-bp direct repeats (Table 3), indicating a duplication at the insertion site. In turn, this not only suggest that it represents an active IS but also that it was recently acquired by the plasmid. It is worth noting that WGS sequence analysis indicated that all the above IS elements are missing in the *A*. *bereziniae* HPC229 chromosome (not shown), strongly suggesting that they were collected during transit of the plasmid through different bacterial hosts.

**Table 3:** Mobile genetic elements detected in pAbe229-114.

Designation	Genetic element	IS Family	Possible origin	Position in plasmid (start-end)	Strand	Length (bp)	Target site duplication	Presence of inverted repeats^a^		Number of copies	Best hit in the ISSaga database^b^	
								IRL	IRR		% nucleotide identity	Accession number
IS*Aba10*	Insertion sequence (IS)	IS*5*	*A*. *baumannii*	4,321–5,343	-	1,023	ATCTAATAC	+	+	1	99	GQ379223
ΔIS*Aba22*	Incomplete IS	IS*3*	*A*. *baumannii*	6,871–7,297	+	427	-	-	+	3	99	CP001937
ΔIS*Aba22*	Incomplete IS	IS*3*	*A*. *baumannii*	102,999–103,153	+	155	-	+	-		99	CP001937
ΔIS*Aba22*	Incomplete IS	IS*3*	*A*. *baumannii*	105,725–106,433	-	709	-	-	+		100	CP001937
IS*Aba2*	IS	IS*3*	*A*. *baumannii*	12,856–14,213	+	1,358	-	+	+	1	91	AY665723
IS*Pa14*	IS	IS*1*	*P*. *aeruginosa*	50,326–51,113	-	788	-	+	+	1	94	KX426227.1
IS*5* family^c^	Defective IS	IS*5*	*A*. *lwoffii*	55,257–56,018	+	762	-	+	+	1	99	KX528687.1
IS*Aba12*	IS	IS*5*	*A*. *baumannii*	58,075–59,113	+	1,039	-	+	+	2	96	NZ_ACYR01000092
IS*Aba12*	IS	IS*5*	*A*. *baumannii*	103,154–104,192	+	1,039	-	+	+		99	NZ_ACYR01000092
ΔIS*Aba34-*like	Incomplete IS	IS*3*	*A*. *baumannii*	78,361–78,745	-	385	-	+	-	1	89	KU744946
IS*Ajo2*	IS	ISNCY	*A*. *johnsonii*	83,347–84,827	+	1,481	-	+	+	1	91	NZ_CP010351.1
ΔIS*Aba1*	Incomplete IS	IS*4*	*A*. *baumannii*	84,870–85,052	-	183	-	-	+	2	99	AY758396
IS*Aba1*	IS	IS*4*	*A*. *baumannii*	106,434–107,613	+	1,180	-	+	+		99	AY758396
IS*Pst3*	IS	IS*21*	*P*. *stutzeri*	95,912–98,516	+	2,605	-	+	+	1	98	AB088753
IS*Abe18*	IS	IS*4*	*A*. *bereziniae*	108,755–110,024	+	1,269	GCTATAGGC	+	+	1	100	CM012183.1
ΔIS*1006*	Incomplete IS	IS*6*	*A*. *junii*	110,050–110,271	-	222	-	+	-	1	100	NC_004361
ΔIS*5* family	Incomplete IS	IS*5*	*A*. *lwoffii*	110,272–110,778	+	507	-	-	+	1	99	KX528687.1
ΔTn*6018*	Transposon		*A*. *baumannii*	56,125–58,074	+	1,960	-	+	-	1	99	FJ172370.5
Tn*6637*^d^	Transposon		*A*. *bereziniae*	84,870–107,603	+	22,744	AATAAAGAT	+	+	1	100	CM012183.1

^a^IRL, inverted repeat left: IRR, inverted repeat right. (+), detected; (-), non-detected.

^b^www-is.biotoul.fr; and [36].

^c^Premature stop codon in the transposase gene.

^d^Tn*6637* is flanked by two IS*Aba1* copies, one incomplete (ΔIS*Aba1*) retaining the IRR.

We also noted that pAbe229-114 bears a region of 18,283 bp (nucleotides 84,870–107,613 in Fig 1) bracketed by incomplete IS*Aba1* and IS*Aba22* elements, which exhibits a higher-than-average GC content (approximately 57%; see inner ring in Fig 1). When considering that the average GC content of *Acinetobacter* genomes is in average around 40% [62], it seems likely that this region was acquired by HGT from a donor from outside the *Acinetobacter* genus. In line with this assumption, a sequence exhibiting a similarly high GC content and displaying more than 85% nucleotide identity was identified in the draft genome sequences of *Pseudomonas stutzeri* B1 SMN1 (accession number AMVM00000000.1), an organism showing an analogous GC content [63]. Moreover, this region is contiguous in pAbe229-114 to a genetic element encompassing a *gshR* glutathione reductase gene (region 11, Fig 1, Table 2) which is surrounded by an IS*Aba12* copy and a defective IS*Aba22* element that is immediately followed in turn by a complete IS*Aba1* copy (Fig 1, Table 3). IS*Aba1* exhibits a large impact on *A*. *baumannii* genomes [61], and therefore we hypothesize that the two IS*Aba1* elements bracketing the 22,744 bp region represent a composite transposon encompassing the oxidative stress resistance genes, the incomplete conjugal-transfer region, and the *gshR* gene. Supporting this notion, the two IS*Aba1* elements at the borders are limited by identical 9 bp-duplications immediately next to their external inverted-repeats, a hallmark of a recent IS*Aba1* transposition event [64]. This novel transposon was thus deposited at the Transposon Registry (https://www.lstmed.ac.uk/services/the-transposon-registry) under the designation Tn*6637* (Table 3). We also identified in pAbe229-114 an incomplete Tn*6018* transposon carrying cadmium and zinc resistance genes (Fig 1). Notably, the Tn*6018* element in pAbe229-114 lacks the *tnpA* transposase gene normally located downstream of the *lspA* gene [55], probably as the result of an IS*Aba12* insertion in this region. Downstream of the *cadR* gene, this defective transposon was additionally found to be interrumped by an IS*5* family element (Fig 1).

Overall the above analysis indicates a mosaic structure for pAbe229-114, most likely the outcome of the sequential insertion of different mobile elements accompanying the plasmid backbone followed by substantial rearrangements and deletions on the resulting structures. These events were most probably selected under varying external conditions during transit through different environmental and clinical bacterial hosts.

### The small HPC229 plasmids pAbe229-15 and pAbe229-9

*A*. *baumannii* clinical strains contain a varied repertoire of plasmids [2,3,6,13,17,29]. Many of them encode only a limited number of functions generally related to mobilization, a TA system, and a replicase of the Rep_3 superfamily, and are designated as “small” plasmids [6]. As detailed below, HPC229 plasmids pAbe229-15 and pAbe229-9 fulfill these traits, and could then be included in this category. pAbe229-15 (15,389 bp, 35% GC content; Fig 2A) contains 16 ORFs, 9 of them encoding proteins of known functions (S2 Table). Among them, replication (RepB), transfer (MobA), stability (a HigB2/HigA2 TA system), and adaptive (a YddG permease (DMT superfamily), a proline/glycine betaine ProP transporter, and a putative creatinase) functions were identified. In the replication module, the replication initiator protein RepB shows 100% amino acid identity with other Rep encoded in different *Acinetobacter* species (WP_012780181.1; S2 Table) including the Rep of pMMD carried by an *A*. *baumannii* clinical strain (YP_006961790.1; S3 Table) [16] and assigned to the AR3G3 group [30]. Four iterons of 21 bp were identified 44 bp upstream of the *repB* start codon (Table 1, Fig 2A), a region showing 100% nucleotide identity with a homologous region also located in pMMD. It is worth noting the presence in the adaptive region of pAbe229-15 of a gene encoding a protein showing both creatinase/prolidase (pfam01321) and creatine amidinohydrolase (pfam01321) domains. These two domains are commonly found in bacterial creatinases responsible for the hydrolysis of creatine to sarcosine and urea, thus providing carbon and nitrogen for growth [65]. The coexistence of genes encoding transporters and a transcriptional regulator belonging to the GlxA family in the same region (S2 Table) suggests that they shared a common metabolic pathway.

BlastN-searches uncovered significant sequence identities between different regions of pAbe229-15 comprising replication, transfer, stability and adaptive functions with those of other *Acinetobacter* spp plasmids (regions 1–5, Fig 2A, and Table 2). The overall analysis thus revealed high levels of identity between different fragments of pAbe229-15 with equivalent regions found among members of the *A*. *calcoaceticus/A*. *baumanni* (ACB) complex.

pAbe229-9 (9,154 bp, 35.4% GC content; Fig 2B) contains 13 predicted ORFs, 7 of them coding for proteins of attributed functions (S2 Table). Its replication region contains a *repB* gene encoding a Rep_3 family protein with 100% amino acid identity with an homologous protein encoded in *A*. *pittii* PR331 (OTM21916.1) and 85% identity with an homologous protein encoded in pAV1 from the environmental strain *A*. *venetianus* VE-C3 (YP_001661463.1; S3 Table). Thus, similarly to the latter protein, pAbe229-9 RepB was assigned to the AR3G6 group following a recent classification [30]. The *oriV* region is composed by four perfect, directly oriented iterons located 56 bp upstream of *repB* (Table 1, Fig 2B). The whole replication region, including *repB* and the iterons, shows homology with a segment of *A*. *venetianus* VE-C3 pAV1 (region 1, Fig 2B, and Table 2). The stability region carries genes for a SplT/SplA TA system [66] and a toxin of the Doc-type. *splT*/*splA* homologous genes were identified also in pAV1 (region 3) and in pD36-4 of *A*. *baumannii* D36 (partial region 4, Fig 2B, and Table 2). The latter plasmid also shares with pAbe229-9 a downstream segment (region 4, Fig 2B), which includes two ORFs of unknown function, and a *doc* gene encoding an orphan toxin of the Phd-Doc system (S2 Table). Concerning the transfer region, a 2,637 bp-homologous segment including a *mobA* gene was found in *A*. *baumannii* pMMD (region 6, Fig 2B, and Table 2). Interestingly, the latter fragment also exhibits 93% nucleotide identity with a partial segment in region 1 of pAbe229-15 (Fig 2A) that spans from nucleotide positions 6,828 to 9,154 and continues from nucleotides 1 to 313 (Fig 2B). This strongly suggested that this region was exchanged in the past between these two HPC229 replicons.

### Phylogenetic analysis of the relaxases encoded by plasmids pAbe229-15 and pAbe229-9

**Fig 3:**
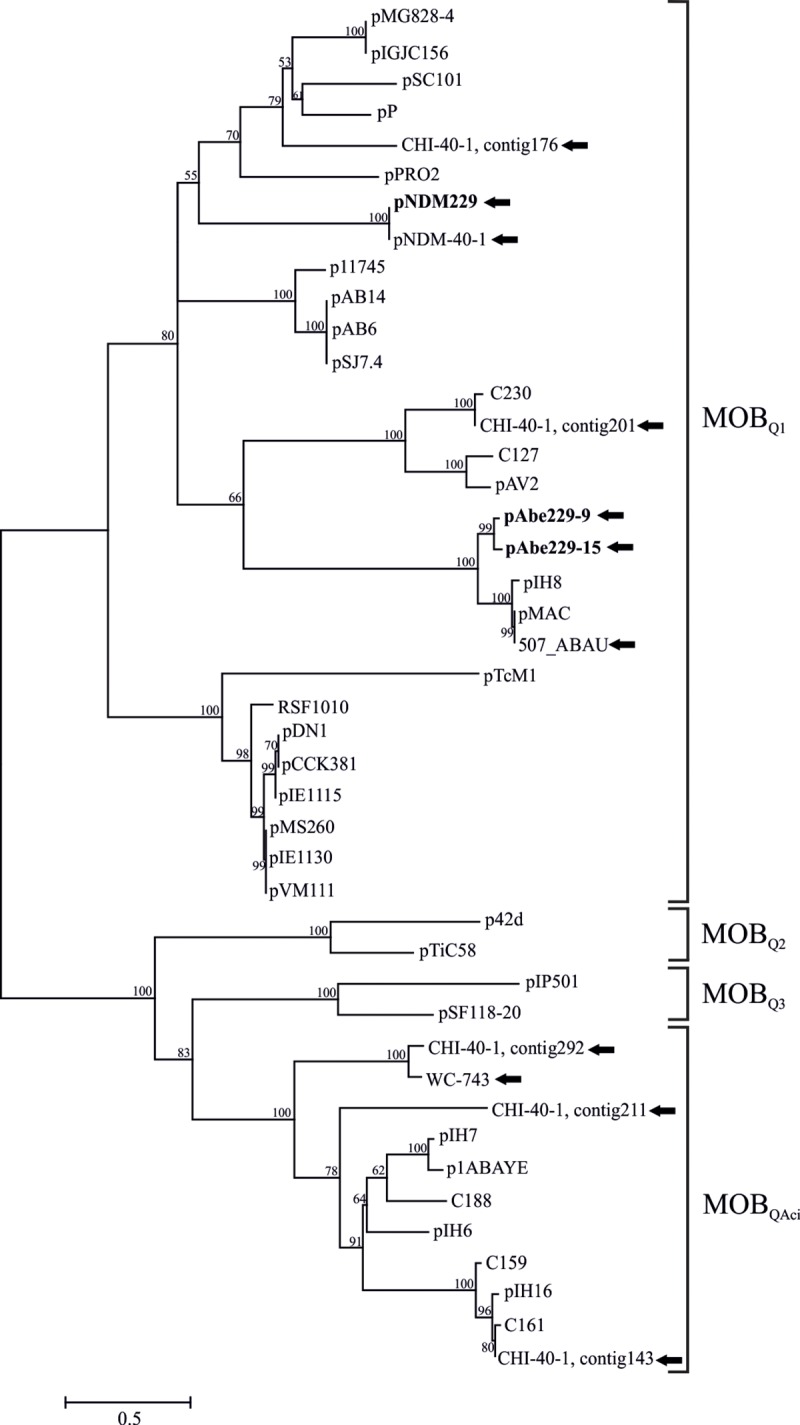
Phylogenetic analysis of HPC229 plasmid relaxases. A ML tree was inferred from the alignments of the first 300 amino acids of the N-terminal domains of MOB_Q_ relaxases from *A*. *bereziniae* and the *Acinetobacter* local database [3,30]. The MOB_Q1_, MOB_Q2_, MOB_Q3_ and MOB_QAci_ sub-families are indicated. Relaxases encoded in *A*. *bereziniae* genomes are specified with black arrows, and those corresponding to HPC229 plasmids are additionally highlighted in bold. In the case of the CHI-40-1 draft genome (GenBank accession number CDEL01000000.1), the contig number in which a given relaxase gene was identified is additionally indicated. The evolutionary scale (number of amino acid substitution per site) is indicated at the botton left. Bootstrap values higher than 50% (100 replications) are indicated at the branching nodes of the ML tree.

The two *mobA* relaxases encoded in pAbe229-15 and pAbe229-9, respectively (S4 Table) were phylogenetically characterized following described procedures [30]. The corresponding N-terminal domain sequences were aligned with other 42 bacterial plasmid relaxases (S4 Table), and a ML phylogenetic tree was subsequently constructed (Fig 3). This analysis revealed that both relaxases clustered with members of the MOB_Q1_ subfamily, in particular within a subclade encompassing the relaxases from *A*. *baumannii* plasmids pMAC and pIH8. On the contrary, the relaxase encoded in pNDM229 [20] clustered within a different MOB_Q1_ subclade (Fig 3). It is worth remarking that in all HPC229 plasmids carrying *mobA* genes the genetic organization of the mobilization region is similar to that described for other *Acinetobacter* plasmids exhibiting a single *mob* gene [67].

### Functional analysis of the transfer regions of pAbe229-114, pAbe229-15 and pAbe229-9

In order to determine whether pAbe229-114, pAbe229-15 and pAbe229-9 transfer regions can be mobilized when providing a full transferability machinery *in trans*, a functional analysis was conducted by cloning first the corresponding regions encompassing *oriT* and *mob* genes (Figs 1 and 2) into pGEM-T-Easy (Promega, Madison, WI, USA) thus generationg pGEM-mob114, pGEM-mob15 or pGEM-mob9. These plasmids were then transformed into *E*. *coli* Eco S17 harboring full conjugation functions [48], and independent mating assays were done using as donors Eco S17 cells harboring pGEM-mob114, pGEM-mob15 or pGEM-mob9, and *E*. *coli* DH5α (rif^R^) as recipient (see Materials and Methods for details). The obtained conjugation frequencies were 1.0 x 10^−6^ UFC/ml, 6.0 x 10^−7^ UFC/ml, and 4.0 x 10^−7^ UFC/ml for pGEM-mob114, pGEM-mob15 or pGEM-mob9, respectively. Of note, when the mating was done under the same experimental conditions employing *E*. *coli* EcoS17 transformed with the empty vector pGEM-T-easy (Promega, Madison, WI, USA) as donor, rif^R^ DH5α transconjugants were not obtained. In turn, no transconjugant cells were obtained when the mating assays were done using rifampicin susceptible *E*. *coli* DH5α harboring pGEM-mob114, pGEM-mob15 or pGEM-mob9 as donors, and rif^R^ *E*. *coli* DH5α as recipient.

In summary, the pAbe229-114, pAbe229-15 and pAbe229-9 regions encompassing *oriT* and Δ*traI* in the case of pAbe229-114, and the corresponding *oriT* and *mobA* in the cases of pAbe229-15 and pAbe229-9, were found to be functional as judged by the above assays. The overall experimental assays thus support bioinformatic predictions indicated in all cases an *oriT* region located upstream genes codifying relaxase proteins involved in horizontal transfer (Fig 1, Fig 2 and S2 Table). This support the notion that *A*. *bereziniae* pAbe229-114, pAbe229-15 and pAbe229-9 can be confidentially classified as mobilizable plasmids.

### The smallest HPC229 plasmids, pAbe229-4 and pAbe229-1, lack rep genes

The presence of small cryptic plasmids lacking known replication initiatior protein genes has been already described in *A*. *baumannii* [6]. Similarly, two of the plasmids found in *A*. *bereziniae* HPC229, pAbe229-4 (4,450 bp, 36.4% GC; Fig 2C) and pAbe229-1 (1,329 bp, 36.9% GC; Fig 2D) fall into this category (S2 Table). pAbe229-4 contains 7 ORFs, 3 of them with described functions including a RelE/RelBTA system and a *rimI* gene encoding a putative N-acetyltransferase. BlastN analysis disclosed significant regions of identity between pAbe229-4 with genomes of other *Acinetobacter* species (regions 3 and 4, Fig 2C, and Table 2).

pAbe229-1, with only 1,329 bp (Fig 2D and S1 Fig), represents the smallest plasmid reported so far in an *Acinetobacter* genus member. It harbors 2 predicted ORFs with no homology in databases, but BlastN homology searches against the nucleotide GenBank database revealed significant homology to a 465 bp region of unknown function found in plasmid pM131-11 from an *Acinetobacter* sp. isolate (Fig 2D, Table 2).

The absence of *rep* genes in pAbe229-4 and pAbe229-1 prompted us to search for alternate replication systems. In pAbe229-4 this analysis indicated the existence of sequences encoding homologous to RNAI (106 bp, between positions 4237 to 4342; Fig 2C) and RNAII (608 bp between position 4236 to 4450 and 1 to 393; Fig 2C), exhibiting 54% and 44% identity, respectively, to the equivalent regions described for ColE1 replicons [68,69]. pAbe229-4 would then represent, to our knowledge, the first example of a ColE1-type plasmid described in a species of the genus *Acinetobacter*. In the case of pAbe229-1, our analysis revealed only a high-AT region typical of a putative replication origin [70] (see potential *oriV* in the GC skew of Fig 2D), but its exact mechanism of replication is still unknown.

In summary, pAbe229-114, pAbe229-15 and pAbe229-9 plasmids encode replication initiator proteins of the Rep_3 superfamily similarly to the case of most other *Acinetobacter* plasmids [6,13,30,71]. This certainly opens the possibility for these three plasmids to replicate in other species of the *Acinetobacter* genus. On the contrary, pAbe229-4 and pAbe229-1 plasmids lack replication initiator protein genes. However, while the pAbe229-4 plasmid may use a ColE1-type replicon as suggested by sequence analysis, the replication mechanism of pAbe229-1 remains obscure.

### Identification of XerC/D sites in HPC229 plasmids

Different authors [13,15–18] have noted the presence in different *Acinetobacter* plasmids of various modules, including antimicrobial resistance genes, heavy metal resistance genes, and TA systems among other genes, bordered by short DNA sequences recognized by XerC/XerD site-specific tyrosine recombinases (XerC/D sites). XerC/D sites are generally composed by two conserved motifs of 11 bp, separated by a less-conserved central region (cr) generally spanning 6 nt [72], although sites with cr regions of five, seven, and even more nucleotides have also been described [14,50,72,73]. The ubiquitous distribution of the XerC/XerD site-specif recombination system among bacteria has led to proposals that the XerC/D sites bordering these modules play roles in their mobilization and dissemination [13,15–18], although the exact mechanism is still obscure [17]. In this context, we have recently found [13] that at least some of the XerC/D sites located in a number of *A*. *baumannii* plasmids can in fact conform proficient pairs for site-specific recombination mediating the formation (and resolution) of plasmid co-integrates. We thus decided to investigate by bioinformatic procedures the abundance and location of XerC/D recognition sites in HPC229 plasmids (see Materials and Methods for details).

This approach allowed us to infer the existence of 12 putative XerC/D sites among 4 of the plasmids present in this strain: 5 in pAbe229-15; 4 in pAbe229-9; only 2 in pAbe229-114; and 1 in pAbe229-4 (Table 4). As previously observed for other bacterial XerC/D sites [13,16–18], the XerD recognition motif in HPC229 plasmids was more conserved than the XerC equivalent. In turn, the cr was the less conserved region both in sequence and in length (Table 4). Thus, while ten of the above 12 XerC/D sites show cr of an usual length of 6 nucleotides (cr6) displaying high sequence variability, one site (XerC/D_8, located in pAbe229-9) contained a cr of 5 nucleotides while other (XerC/D_5, located in pAbe229-15) a cr of 7 nucleotides in length (Table 4).

**Table 4:** XerC/D sites in HPC229 plasmids.

Plasmid	XerC/D-site^a^	Nucleotide sequence^b^			cr length (bp)	Position in plasmid (start-end)	Search method^c^
		XerC	cr	XerD			
pAbe229-15	XerC/D_1	···········	··c···	···········	6	2,174–2,201	*
	XerC/D_2	g··········	cc····	···········	6	6,904–6,931	*
	XerC/D_3	·c···tc····	·a·a·t	···········	6	9,477–9,504	*
	XerC/D_4	·c···tc····	·a·a·t	···········	6	10,204–10,231	*
	XerC/D_5	·a··aac····	tac·c·a	·····cg····	7	13,062–13,090	**
pAbe229-9	XerC/D_6	g··········	c··cc·	···········	6	3,622–3,649	*
	XerC/D_7	·c····c····	··t···	·········t·	6	4,473–4,500	*
	XerC/D_8	·····a·····	atca·-	····a·gg··a	5	6,778–6,804	**
	XerC/D_9	···········	·a·a·t	·········g·	6	6,828–6,855	*
pAbe229-114	XerC/D_10	···········	··t···	·········t·	6	59,663–59,690	*
	XerC/D_11	taac··a····	c·ccc·	·········t·	6	82,467–82,494	**
pAbe229-4	XerC/D_12	···········	c·t·c·	·········t·	6	2,889–2,916	*
	**consensus**^**d**^	**atttCgtATAA**	**ggggta**	**TTATGTTAAaT**	** **		

^a^The numbers assigned to the different XerC/D-like recognition sites in HPC229 plasmids were arbitrarily chosen.

^b^In each of the inferred XerC/D sites the presence of the same nucleotide in a given position is denoted with a dot sign (.), otherwise the corresponding nucleotide is indicated in lowercase letters; cr: central region.

^c^XerC/D sites in HPC229 plasmids were detected by using Fuzznuc (http://www.bioinformatics.nl/cgi-bin/emboss/fuzznuc) using as query NNTNYKYATAANNNNYWTTATSTKAWNN (*) [13], or by visual inspection (**).

^d^New consensus inferred from the XerC/D-like sites identified in HPC229 plasmids. Only XerC/D sites displaying a cr of 6 nucleotides in length were considered for this purpose.

The above XerC/D sites were bracketing discrete regions in the corresponding HPC229 plasmid sequences (Figs 1 and 2). BlastN-homology searches using as query each of these regions indicated high levels of identity with similar regions carried by different *Acinetobacter* plasmids for some of them, including region 1 and 2 of pAbe229-15 (Fig 2A, Table 2), and region 3 of pAbe229-9 (Fig 2B, Table 2). It is worth noting also that a region of around 1 kbp in pAbe229-114, that includes the XerC/D_11 site and a downstream *higA2*/*higB2* TA system (region 9, Fig 1) showed high sequence identity with a homologous segment found in pOXA58-AP_882 of *A*. *pittii* AP_882 (Table 2). This TA system is bracketed by a XerC/D pair in the latter plasmid (CP014479.1), thus suggesting that it may have been acquired by pAbe229-114 through XerC/D-mediated site-specific plasmid fusions followed by further plasmid rearrangements [13].

In summary, the presence of several XerC/D recognition sites in many of HPC229 plasmids, and the modular nature of the sequence regions that they border (see above) are consistent with the idea that XerC/D-mediated site-specific recombination events may have played relevant roles in the evolution of their structures [13].

### Analysis of plasmid idiosyncratic sequences (plasmid markers) in *A*. *bereziniae* genomes

To obtain further clues on the diversity of the plasmids housed by the *A*. *bereziniae* population, we conducted a comparative genomic analysis of genome sequences obtained from all strains assigned to this species available at GenBank (NCBI) database. For this purpose, we retrieved and analysed the available genomic sequence data corresponding to all seven strains classified as *A*. *bereziniae* in the database (S5 Table, top seven strains). We also conducted a BlastN search among the currently available *Acinetobacter* sp. genome sequences (GenBank-WGS database) using as query the *A*. *bereziniae* type strain CIP 70.12 *rpoB* gene sequence (GenBank accession number APQG01000052.1) to identify potential *A*. *berezeniae* strains not assigned to this species. Two strains showing *rpoB* sequences identities higher than 99% with CIP 70.12 *rpoB* emerged from this analysis, *Acinetobacter* sp. Ag2 and *Acinetobacter* sp. WC-743 (S5 Table), which prompted us to further delimitate their species assignations. We therefore calculated the percentage of average nucleotide identities (ANI) of these two strains when compared to the *A*. *bereziniae* type strain CIP 70.12 [23]. We also included in these comparisons other 6 strains previously assigned to *A*. *bereziniae* by other authors (S5 Table). As seen in this Table, ANI values higher than 98% were obtained for all of the 8 strains, largely exceeding in all cases the 95% cut-off value adopted for species assignation among the *Acinetobacter* genus [43]. In comparison, two *Acinetobacter* non-*bereziniae* strains that emerge closely-associated to *A*. *bereziniae* in phylogenetic trees such as *A*. *guillouiae* CIP 63.46 [23] and *A*. *gerneri* DSM 14967 [26] showed ANI values of 83 and 76%, respectively (S5 Table), thus validating the species assignations made above.

Plasmid contigs detected in the above *A*. *bereziniae* strains were first identified on the basis of the *rep* genes they encode. Hence, a list of Rep sequences was manually compiled based on the genomic annotation of all *A*. *bereziniae* strains under study (S5 Table), and in which proteins containing the chromosomal DnaA domain that activates initiation of DNA replication in bacteria (pfam00308) were excluded. Each of the identified Rep was used as query in a BlastP-homology search against a local protein database that included all the *Acinetobacter* replication initiator proteins of the Rep_3 superfamily (AR3) [30]. Sixteen out of the seventeen Rep proteins thus identified among *A*. *bereziniae* sequences fell into 11 of the 15 groups defined in this work [30], including AR3G1.1, AR3G1.2, AR3G1.4, AR3G2, AR3G3, AR3G5, AR3G8, AR3G9, AR3G11, AR3G12, AR3G13, AR3G14, and AR3G15 (S3 Table). A notable case was represented by *A*. *bereziniae* CHI-40-1, in which 7 Rep candidates were found distributed among 7 different AR3G groups (S3 Table). This supports the proposed existence of several different plasmids in this strain [22].

The remaining Rep candidate found in an *A*. *bereziniae* strain, KCTC 23199 (GenBank accession number WP_010591570.1; S3 Table), showed no significant identity to any of the above Rep_3 superfamily members. Moreover, it encompassed in the protein sequence an N-terminal replicase domain (pfam03090 family), a primase C-terminal 1 domain (pfam08708 family, PriCT_1) and a helix-turn-helix_28 domain (pfam13518 family). Further BlastP comparative searches against the GenBank protein database identified homologues also sharing these three domains not only among other *Acinetobacter* plasmids (S3 Table) but also enterobacterial plasmids including a *Klebsiella pneumoniae* plasmid (47% identity, 99% coverage, GenBank accession number SSI90041.1).

Two Rep proteins, encoded in *A*. *bereziniae* CIP 70.12 and KCTC 23199 sequences, were found to be identical to RepC (WP_000743064.1) codified in plasmid pAB3 of *A*. *baumannii* ATCC 17978 (S3 Table), which was assigned to AR3G14 [30]. However, these proteins contain a RepC (pfam06504) rather than a Rep_3 (pfam01051) superfamily domain, and were thus assigned to a novel group tentatively denominated ARCG1 (S3 Table). RepC superfamily members are found among IncQ-like group plasmids [74] where they function as iteron-binding *oriV* activators [75]. It follows that *A*. *bereziniae* is seemingly capable of hosting a wide variety of plasmids, the majority of them containing Rep_3 based replication modules.

We also analyzed the putative relaxases encoded in *A*. *bereziniae* sequences by undertaking the same phylogenetic approach used above for pAbe229-15 and pAbe229-9 (Fig 3, S4 Table). Eight proteins annotated as relaxases in *A*. *bereziniae* genomes were also included in this analysis with strain CHI-40-1 bearing 5 candidates, one of them in pNDM-40-1 (AHF22521.1; S4 Table) [22]. Our analysis (Fig 3) indicated that 4 out of these 8 proteins clustered within MOB_Q1_ together with the 3 relaxases detected among HPC229 plasmids, and the remnant 4 proteins clustered with members of the recently described MOB_QAci_ subfamily [30].

In summary, the wide repertoire of both replication and mobilization functions described above among *A*. *bereziniae* plasmids, and especially among strains HPC229 and CHI-40-1, reflect the plasmid diversity housed by this species.

### Comparative sequence analyses between HPC229 plasmids and other *A*. *bereziniae* genomes

**Fig 4:**
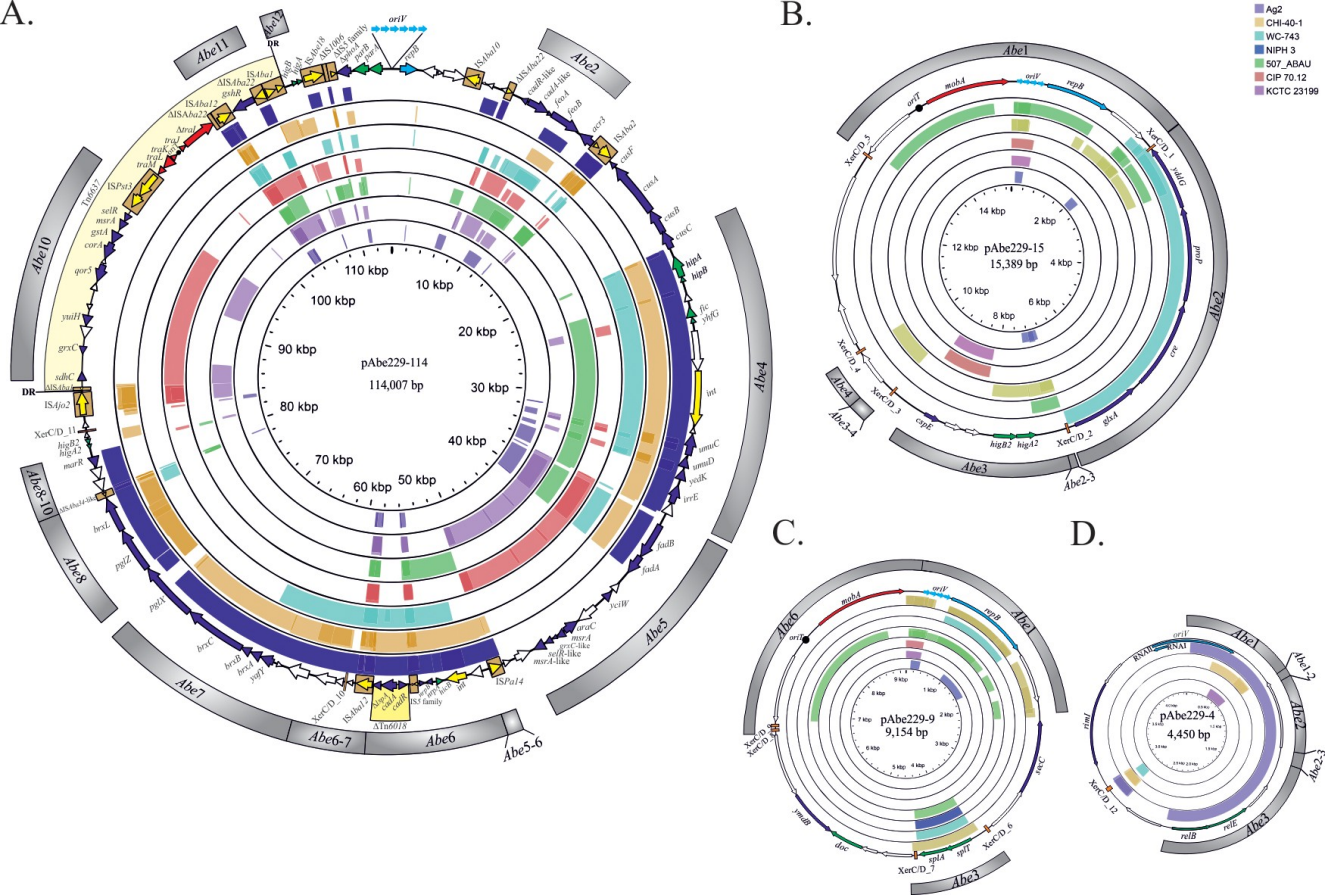
Identification of homologous sequences to HPC229 plasmids in other Acinetobacter genomes. The colored arrows in the outer ring describe the location, identification, and direction of transcription of genes in the corresponding plasmids with described functions in databases. ORFs encoding for unknown functions are indicated by open arrows. In the subsequent rings, the regions of homology between the corresponding plasmid sequences and genome sequences of other A. bereziniae strains (indicated by arcs of different colors, see key near the upper right margin) as detected by BlastN searches are shown. The strains have been located from the highest (outer rings) to the lowest (inner rings) sequence coverage. The height of the colored arcs in each case is proportional to the percentage of nucleotide identity obtained in the BlastN search. BlastN hits with ≥70% nucleotide identity and a minimum alignment length of 1,000 bp (pAbe229-114) or 300 bp (pAbe229-15, pAbe229-9 and pAbe229-4) are detailed in Table 2. The darker lines in the arcs mark overlapping hits. The external Abe1-Abe12 grey regions indicate homologous sequences found in Acinetobacter non-bereziniae genomes (For details see Figs 1 and 2; and also Table 2).

We next analyzed whether the plasmids identified here in HPC229 share significant regions of identity with sequences of other *A*. *bereziniae* genomes (S5 Table, Fig 4). We used for this purpose the draft genome sequences from 7 of the above indicated *A*. *bereziniae* strains, and excluded from the analysis strain XH901 since its reported genome contains only chromosomal sequences. This analysis indicated that the genomic island described in pAbe229-114 (region Abe4, Fig 4A) was also detected in other *A*. *bereziniae* strains (see also Table 2; results obtained for sequences present in *A*. *bereziniae* genomes are shown in the lower part). The presence of this genomic island among these genomes suggests that it may have been acquired by HGT, although its presence in an *A*. *bereziniae* ancestor followed by differential losses in some lineages cannot be ruled out at this stage.

Other regions that deserve consideration in pAbe229-114 are those encoding oxidative stress resistance mechanisms (Abe5 and Abe10 regions, Fig 4A), heavy metal (cadmium, cobalt, nickel and zinc) detoxification systems (Abe6 region, Fig 4A) and type 1-BREX system (Abe7 and Abe8 regions, Fig 4A, Table 2). All corresponding genes were also identified in other *A*. *bereziniae* strains (Table 2). It is worth noting that similar systems bearing strong adaptive significance have been described in plasmids from both pathogenic species of the genus such as *A*. *baumannii* [76,77] as well as from different environmental microbial populations [18,78]. Taken together, the above data reinforce the notion that these adaptive traits are being disseminated by plasmids among different bacterial populations. Moreover, the Abe12 region bearing *higB/higA* genes is also present in several *A*. *bereziniae* strains. Notably, the genes encoding all of these elements are contained in contig143 of strain CHI-40-1. This contig also harbors a *mobA* gene (Fig 3), thus suggesting a plasmid location of this TA system and supporting the above proposal that these systems are frequently mobilized by plasmids [79]. Altogether, the above comparisons indicate that *A*. *bereziniae* strains isolated in different geographical locations (S5 Table) share between them a significant number of DNA regions, most of them likely encoding adaptive functions. Still, pAbe229-114 carries a unique set of replication, stability and transfer genes as compared to other *A*. *bereziniae* strains (Table 2). This suggests a high genetic plasticity for this species and the capability to mobilize DNA segments exhibiting adaptive functions among different *Acinetobacter* replicons.

Comparative sequence analyses done on pAbe229-15 (Abe1-Abe4 regions, Fig 4B, Table 2) showed homology to sequences present in other *A*. *bereziniae* genomes, with the exception of the region encompassing nucleotides 10,236–13,127 encoding two ORFs (orf13 and orf14) of unknown functions. Notably, the Abe3-4 and Abe4 regions flanked by XerC/D_3 and XerC/D_4 sites shows 98% sequence identity with an homologous region from *A*. *bereziniae* CHI-40-1 contig195 (Fig 4B, Table 2). This segment in CHI-40-1 also encodes a Rep_3 member (CDEL01000195.1; Table 2), thus suggesting that this contig corresponds to a plasmid. Thus, it is tempting to speculate that the Abe3-4 and Abe4 regions are shared by plasmids present in both CHI-40-1 and HPC229, and that are probably mobilized by recombination. Interestingly, the unique region of pAbe229-15 is flanked by sites XerC/D_4 and XerC/D_5 (Fig 4B), also suggesting an acquisition as the result of site-specific recombination.

In the case of pAbe229-9, a region carrying the *splT/splA* genes (Abe3 region; Table 2) displayed significant identity with sequences derived from several *A*. *bereziniae* strains (Fig 4C). This region, also present in several *A*. *bereziniae* genomes, is bracketed by XerC/D_6 and XerC/D_7 sites. Moreover, the presence of a similar arrangement (including the bordering XerC/D sites) in pAV1 of *A*. *venetianus* VE-C3 also suggests a mechanism of acquisition of this TA encoding region mediated by site-specific recombination [13].

Regarding pAbe229-4, an homologous region of 2,352 bp including orf1, orf2, and the *relEB* TA system (Abe1, Abe1-2, Abe2, Abe2-3 and Abe-3, Fig 4D) was only detected in *A*. *bereziniae* Ag2 (Table 2). Finally, pAbe229-1 homologous sequences could not be detected among *A*. *bereziniae* genomes other than HPC229.

Altogether, the above observations suggest that HPC229 plasmids are specific of *A*. *bereziniae* HPC229, since none of them showed significant coverage with sequences present in other *A*. *bereziniae* strains. Still, it is worth noting that 4 out of 5 HPC229 plasmids displayed homology with sequences present in strain CHI-40-1 including stretches of 42,386 bp (pAbe229-114), 2,571 bp (pAbe229-15), 1,422 bp (pAbe229-9), and 444 bp (pAbe229-4) (see total cover column, Table 2). Furthermore, the modules conforming the HPC229 plasmid backbone are not widely represented in other members of this species, suggesting they were partially acquired from *A*. non-*bereziniae* species by HGT.

### XerC/D sites in *A*. *bereziniae* genomes

The presence of XerC/D recognition sites in *A*. *bereziniae* genomic sequences was also investigated. In principle we expected to detect the chromosomal cognate site (*dif*) involved in chromosome segregation [72], so we hypothesized that the additional XerC/D sites detected in a given genome should be carried by plasmids. To search for XerC/D sites among these sequences, a partially degenerate 28-nucleotides consensus query bearing a 6 nucleotides central region (cr), as inferred from HPC229 plasmid (Table 4), was used (see Materials and Methods for details). Moreover, and given the lack of conservation in the cr on the consensus sequence mentioned above, we additionally modify the query by including a completely degenerate cr in this part of the sequence.

A total of 55 putative XerC/D sites were identified by this procedure in the eight *A*. *bereziniae* strains analyzed (S6 Table). Expectedly, only one XerC/D site was identified in strain XH901 lacking plasmid sequences (see above), probably reflecting the single chromosomal *dif* site. In most of the other strains the number of XerC/D sites varied between 2 and 7, with the exception of strains HPC229 (10 sites, Table 4) and CHI-40-1 (31 sites, S6 Table). This is in line with the above observations indicating that the latter two strains carry several plasmids, and suggests a correlation between strains adapted to the hospital environment and the presence of multiple XerC/D sites in the plasmids they carry [13].

With the information obtained above from all the 67 *A*. *bereziniae* XerC/D sites, we constructed a atttCgcATAAggggtaTTATGTTAAaT XerC/D consensus recognition site (S6 Table, see also Table 4). This consensus resulted very similar to that reported previously for the plasmids present in the *A*. *baumannii* Ab242 clinical strain (atTtcgtATAAggtgtaTTATgTtAaat) [13] with the exception of two mismatches, one at the XerC site and another at the cr. This opens the possibility of XerC/D-mediated site-specific recombinatorial events between plasmids of both species if they eventually come in contact within the same bacterial host.

## Conclusions

A detailed characterization of the *Acinetobacter* accessory genome can provide clues on the evolutionary dynamics of the members of this genus. In this context, we analyzed here the plasmid content of *A*. *bereziniae* HPC229, being this species isolated from various human, animal and environmental sources [23]. This clinical strain represents, to our knowledge, the first *A*. *bereziniae* strain in which a complete and diverse pool of plasmids has been characterized. The ability of *A*. *bereziniae* HPC229 to harbor six plasmids, including pNDM229 carrying *bla*_NDM-1_ and *aphA6* resistance genes most likely selected in the clinical setting [20] as well as the other plasmids characterized here probably enabling environmental survival, provides this strain with a great plasticity to thrive under various selective pressures. The overall analysis suggest that *A*. *bereziniae* HPC229 plasmids could be regarded as chimeras of diverse origins. An outstanding example is pAbe229-114, which shares extensive backbone sequence similarity to pAV3 from *A*. *venetianus* VE-C3 isolated from polluted waters [80]. This correlation was not totally unexpected, providing that *A*. *bereziniae* is also isolated from waste water products of human activities [26].

The small HPC229 plasmids pAbe229-15 or pAbe229-9 show features which may contribute to their dissemination by HGT. These include MOB_Q1_ relaxases, which could be assisted by helper plasmids from different incompatibility groups and/or chromosomally-located *tra* functions [67]. Interestingly, these small plasmids do not carry mobile elements but are enriched in XerC/D-recognition sites bracketing specific regions, which could mediate the formation of cointegrates with other XerC/D-containing plasmids by site-specific recombination [13]. In this context, our analysis of *A*. *bereziniae* genome sequences suggests a correlation between strains adapted to the hospital environment such as HPC229 and CHI-40-1 [20,22], and the presence of multiple XerC/D sites in many of the plasmids they carry.

In summary, our studies highlight the plasmid diversity carried by *A*. *bereziniae*, an organism that may contribute to the evolution and expansion of the *Acinetobacter* plasmidome facilitating thus the adaptation of other species from this genus to these radically distinct environments.

## Supporting information

S1 Fig S1 nuclease analysis of HPC229 plasmids. Plasmids extracted from *A*. *bereziniae* HPC229 without (lane 1) or with S1 nuclease treatment (lane 2) were resolved by agarose gel electrophoresis. The linearized forms of pAbe229-15, pAbe229-9, pAbe229-4 and pAbe229-1 are highlighted by black triangles. The final positions of the size markers (EcoRI/HindIII-digested Lambda DNA) are shown at the left margin.
(TIF)

S1 Table Oligonucleotide primers used for PCR analysis. (PDF)

S2 Table Genes identified in HPC229 plasmids. (XLSX)

S3 Table *A. bereziniae* plasmid replicases classification. (XLSX)

S4 Table Plasmid relaxases used in this work. (XLSX)

S5 Table Characteristics of *A. bereziniae* and type strains. (XLSX)

S6 Table XerC/XerD sites in *A. bereziniae* genomes. (XLSX)
