# Supplementary material for: Characterization of the diverse plasmid pool harbored by the blaNDM-1-containing Acinetobacter bereziniae HPC229 clinical strain
Source: PLoS One. 2019 Nov 19;14(11):e0220584. doi: 10.1371/journal.pone.0220584 (PMC6863613; doi:10.1371/journal.pone.0220584)
Supplement: S1 Table — (PDF) [file pone.0220584.s002.pdf]

Table S1. Oligonucleotide primers used for PCR analysis

Plasmid	Primer pairs[a]		Hybridization region in each plasmid	Sequence (5´→3´)	Expected product size (bp)	Accession number
pAbe229-114	A	91-F	59,292-59,312	CTGCATGTACAAACTGTTCAC	947	CM012183.1
		37-R	60,235-60,254	GATCCACGCATAGATACAGG		
	B	29-F	56,035-56,054	TGGATTATGAGCACCCTAAG	1,178	
		91-R	57,213-57,232	TATGTCCAACCGCATAAAGG		
	C	mob114-F	100,329-100,349	GATCAATCGGCTTTGTATGCG	2,682	
		mob114-R	102,991-103,010	CAGCACACATCATCCTGACC		
pAbe229-15	D	76-F1	10,460-10,478	GCTGGTGATACTGAAGAAG	1,017	CM012184.1
		80-R1	11,458-11,476	GGTTAGAGAGCCAGAGTTG		
	E	80-F2	1,861-1,880	CAGGATATAGCAACGGATCC	1,236	
		76-R2	3,094-3,112	CTCTCCCTGCACTCAATAG		
	F	mob15-F	12,942-12,961	CATGTGTGCAAGCACAAGCG	2,803	
		mob15-R	336-355	GAAAGGCTTGCTCCATAAGG		
pAbe229-9	G	75-F	2,107-2,126	CGTGGAACGTGAATAGTCAC	1,053	CM012186.1
		75-R	3,140-3,159	CTATCGTGGTGATGCTGAAC		
	H	mob9-F	6,644-6,663	GACCATCCCCATTCATAGCC	2,562	
		mob9-R	32-52	AGCGGTTTTTCAGAGGGCTG		
pAbe229-4	I	82-F	3,903-3,921	CAGCTAGCTCCAAGTAGTG	1,013	CM012185.1
		82-R	446-465	CATCTTAGATCGAGCTTTGC		
pAbe229-1	J	95-F	673-692	CGTAGTGATGCCTTACATTG	959	CM012182.1
		95-R	284-302	GCAGGCTACATAACTTTCG		

[a]Specific primers designed for HPC229 plasmids.
